# Developing a new diagnostic algorithm for human papilloma virus associated oropharyngeal carcinoma: an investigation of HPV DNA assays

**DOI:** 10.1186/s40463-017-0189-z

**Published:** 2017-02-13

**Authors:** Natasha Cohen, Michael Gupta, Lilian Doerwald-Munoz, Dan Jang, James Edward Massey Young, Stuart Archibald, Bernard Jackson, Jenny Lee, Max Chernesky

**Affiliations:** 1 Department of Surgery, McMaster University, Hamilton, ON Canada; 2 Department of Surgery, St Joseph’s Healthcare, Hamilton, ON Canada; 3 Department of Radiation Therapy, Juravinski Cancer Centre, Hamilton, ON Canada; 4 Department of Microbiology, St Joseph’s Healthcare, Hamilton, ON Canada

**Keywords:** Human papilloma virus, Tonsillar cancer, Oropharynx, Diagnostic algorithm

## Abstract

**Background:**

Human papilloma virus (HPV) has been implicated in the development of a large proportion of oropharyngeal squamous cell carcinoma (OPSCC). Current techniques used to diagnose HPV etiology require histopathologic analysis. We aim to investigate the diagnostic accuracy of a new application non-histopathologic diagnostic tests to help assist diagnosis of HPV-related oropharyngeal tumors.

**Methods:**

Patients with OPSCC with nodal metastasis were consecutively recruited from a multidisciplinary cancer clinic. Appropriate samples were collected and analyzed. The various tests examined included COBAS® 4800, Cervista® HR and Genotyping. These tests were compared to p16 staining, which was used as the diagnostic standard. StataIC 14.2 was used to perform analysis, including sensitivity, specificity and receiver operator characteristic [ROC] curves.

**Results:**

The COBAS® FNA (area under ROC 0.863) and saliva (area under ROC 0.847) samples performed well in diagnosing HPV positive and negative tumors. Samples tested with Cervista® did not corroborate p16 status reliably. We were able to increase the diagnostic yield of the COBAS® FNA samples by applying the results of the saliva test to negative FNA samples which correctly identified 11 additional p16 positive tumors (area under ROC 0.915).

**Conclusion:**

Surrogate testing for HPV using alternate methods is feasible and closely predicts the results of standard diagnostic methods. In the future, these could minimize invasive procedures for diagnosing HPV-related oropharyngeal cancer, but also help to diagnose and treat patients with unknown primaries.

## Background

Human papilloma virus (HPV) infection is linked to the development of several human malignancies, notably oropharyngeal squamous cell carcinoma (OPSCC) within the head and neck region [1, 2]. In fact, OPSCC has been increasing in prevalence despite decreasing trends in other common cancers [3], and HPV, particularly subtype 16, is thought to contribute to this trend, with over 60% of OPSCC expressing HPV DNA or its markers [1, 4]. HPV related OPSCC has been shown to affect a younger population and is more likely to present with advanced nodal disease but early T-staging [5], and is overall associated with an increased survival rate and improved overall prognosis [6–8]. However, treatment of OPSCC can have significant morbidity including, but not limited to, chronic pain and dysphagia, making it important for diagnostic tests to be developed and studied for application in screening purposes. In fact, to this day there are no widely available or approved diagnostic tests available to help identify patients at risk of OPSCC or with presence early OPSCC.

The goal of this study is to identify a diagnostic algorithm that could be used to predict a patient’s HPV-positivity using surrogate markers. These tests were performed on samples obtained from patients with known OPSCC, as part of a phase 1 diagnostic test study that will identify possible candidates for future use in screening. We also explore a diagnostic algorithm that may help differentiate HPV-positive from HPV-negative malignancy in the presence of known nodal metastatic disease.

## Methods

This study was approved by the Hamilton Integrated Research Ethics Board. Patients were enrolled from a multidisciplinary head and neck cancer clinic, where newly diagnosed biopsy proven OPSCC participants were recruited into the study from July 2012 to July 2015. Patients were considered eligible if they newly diagnosed, histopathologically proven OPSCC with at least one positive lymph node that could be sampled using fine needle aspiration biopsy (FNA). All patients were recruited after signed informed consent.

FNA samples were obtained by senior head and neck surgeons (MG, BSJ, SA, JEMY) for the purposes of the study, and saliva samples and oropharyngeal swabs (tongue base and tonsil) were collected by our research liaison (LDM). If the patients had not yet undergone panendoscopy and multiple biopsies in their work up, FNA was performed intraoperatively at the time of this procedure in order to minimize patient discomfort. The technique for FNA sampling was with multiple passes (greater than 3) into the palpable node using a 22 gauge needle on a 10 cc syringe. For lymph nodes that could not be easily sampled when guided by palpation, ultrasound-guided FNA was performed at the radiology department at St-Joseph’s Hospital in Hamilton. Saliva samples and oropharyngeal swabs collected by our research liaison (LDM), who counselled the participants to spit into a test tube container (roughly 1 ml sample obtained) and collected the swabs by firmly touching both tonsils and accessible oropharynx with the applicator through the oral opening. These samples were then tested for presence of HPV at the Infections Research Laboratory at St-Joseph’s Hospital. The saliva, swabs, and FNA samples were tested for high-risk HPV Cobas® 4800 (Roche, Basel Switzerland), and the FNA and swab samples were tested with Cervista® HR and Cervista® HPV genotyping (Hologic, WI, USA). Results were compared to p16 staining of the primary tumor site. Staining for p16 occurred at the immunohistochemistry lab, and were considered positive if greater than 70% of the primary site tissue biopsy stained for p16. All testers of collected samples were blinded from cytologic and histopathologic results.

### Statistical analysis

StataIC version 14.2 (TX, USA) was used to perform statistical analyses. Receiver operating characteristics (ROC) was used to compare the diagnostic accuracy of the various assays (Cobas® 4800, Cervista HR and HPV genotype) on each sample collected (saliva, oral swabs, FNA). ROC analysis was designed as plot of sensitivity (y-axis) versus 1 minus specificity (x-axis). Results were coded as categorical variables (i.e. test was either positive or negative). Sensitivities, specificities and areas under the ROC were calculated and are reported with 95% confidence intervals (95% CI).

## Results

Of 91 patients enrolled in the study, seventy-seven patients met our inclusion criteria. Seventy participants were male and seven were female. The mean age was 59.4 (SD 9.6, range 40 to 80). There were 66 HPV positive tumors and 11 were HPV negative as confirmed by p16 positivity (HPV positivity 85.7%). Several samples lacked sufficient volume for testing with COBAS® (FNA 6, BOT 1, Saliva 3). As well, Cervista® HR and Genotype was only applied to samples from 43 participants due to limitations related to cost redistribution favoring other tests with higher diagnostic yield. Data from 37 participants met inclusion criteria for our analysis.

Results are summarized in Table 1. COBAS® assays performed best when testing material collected by FNA and saliva. COBAS® testing of FNA was 86% sensitive [ROC area 0.86 (95% CI 0.76–0.93)] and saliva testing being 100% sensitive [ROC area 0.85 (95% CI 0.75–0.92)]. These samples outperformed those collected by swabbing of the oropharynx (OP swab) (ROC area 0.63; 95% CI 0.52–0.74). Cervista® HR and Genotyping assays was not successful in correctly identifying HPV in samples collected by FNA and oropharyngeal swab. Only Cervista® HR oropharyngeal swabs performed better than a coin toss with an ROC area of 0.67 (95% CI 0.45–0.86).

**Table 1:** Assay performance by type of sample

Test	Sample type	Sensitivity	Specificity	Area under curve (ROC)
Cobas® DNA	FNA	91%	82%	0.86 (95% CI 0.75–0.93)
	OP Swab	91%	36%	0.63 (95% CI 0.55–0.75)
	Saliva	100%	69%	0.85 (95% CI 0.75–0.92)
Cervista® HR	FNA	0%	90%	0.45 (95% CI 0.26–0.62)
	OP swab	100%	33%	0.67 (95% CI 0.45–0.86)
Cervista® HPV Genotype	FNA	0%	86%	0.43 (95% CI 0.25–0.61)
	OP Swab	100%	14%	0.57 (95% CI 0.34–0.77)

*HPV* Human papilloma virus, *DNA* Deoxyribonucleic acid, *FNA* Fine needle aspirate, *OP* Oropharyngeal

**Fig. 1:**
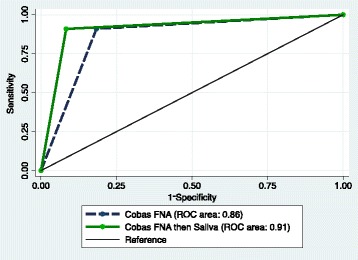
Comparison COBAS® FNA alone and FNA then Saliva

In the second part of the analysis, we studied combinations of these diagnostic tests in order to develop an algorithm that most accurately identified HPV positive tumors. The highest performing tests (ROC greater than 0.8) were tested in a forward stepwise fashion to formulate an algorithm that best predicted HPV status of the primary tumor. Fine needle aspirate Cobas® DNA samples were used as the primary test followed by Cobas® DNA saliva analysis of saliva samples in patients testing negative for HPV with the FNA samples. This combination had a sensitivity and specificity of 91 and 92% respectively, and an area under the ROC of 0.92 (95% CI 0.83–0.97, see Fig. 1). This combination correctly classified an additional 11 participants.

## Discussion

This study shows promising results for several commercially available tests for diagnosis of HPV-related malignancy of the oropharynx. In fact, we were able to show that the Cobas® 4800 system was most sensitive to HPV positivity when testing FNA and saliva samples. As well, we were able to formulate a simple diagnostic algorithm consisting of FNA testing with COBAS® 4800 on FNA samples followed by Cobas® 4800 assay of saliva samples for those initially testing negative. This simple algorithm yielded an excellent diagnostic accuracy (AUC 0.92).

This study has identified a test that correctly diagnosed HPV positive and negative patients in 92% of HPV-related OPSCC. In fact, the Cobas® 4800 assay when used sequentially on FNA samples followed by saliva sample. This is the first study to our knowledge to assess multiple genetic and molecular tests on various biologic samples obtained from the oropharynx and nodal disease, in a population where HPV positivity can be confirmed by gold standard oncologic testing of primary tumor site.

The diagnostic algorithm we were able to develop is of particular interest in the setting of cancers of unknown primary (CUP). CUPs are defined as the presence of malignancy in one or more lymph nodes in the absence of a primary site malignancy [9]. Although these malignancies present a diagnostic challenge, literature suggests that CUP is related to HPV-infection in a third of cases or more [10–12]. The proposed mechanism for CUP of the head and neck is that the presence of cancer may exist in biopsied areas such as tonsils and may never be identified due to a small foci of cancer that may be missed on pathological assessment, or may result from primary tumor site regression [13] after nodal metastasis has already occurred. Due to the improved prognosis of HPV-related tumors, as well as the future direction of de-escalation therapy [14, 15] in this patient population, establishing a way to correctly identify HPV-related CUP is essential. Furthermore, given the fact that HPV positivity strongly suggests involvement of the oropharynx, knowledge that a cancerous lymph node contains HPV related disease may both aid in the search of occult primaries and help tailor treatment of these cancers.

The head and neck literature has demonstrated the use of assays validated on genital or cervical specimens [16], as well as the use of cytologic brushes of pharyngeal mucosa to confirm oncogenic HPV infection [17, 18]. However, none of these are yet approved for use in determining HPV status in the oropharynx. The use of the swab and saliva-based tests may offer another way to determine high-risk HPV infection.

Limitations of our study include a small sample size due to the fact that this is a single center study, which limits the overall generalizability of the results. However, the goal of this project is of an exploratory nature, therefore the results can be used for sample size calculations as well as guide future research on this topic. Another limitation of our study was due to limited resources, as we were not able to complete testing of Cervista® HR and HPV genotyping on all the samples collected, This limits our ability to determine the diagnostic accuracy of this assay on samples obtained in patients with OPSCC. As well, our analysis was limited by insufficient sample material available for testing, particularly for the Cervista® assays. One case (COBAS® testing of FNA sample) yielded an indeterminate result due to contamination with blood (see Table 2). Due to these limitations, we recommend further studies to better determine the diagnostic accuracy of the tests evaluated here. Finally, the prevalence of HPV positive patients was high in our study (85.7%), likely owing to the inclusion requirement of nodal disease at presentation. However, this should not impact the results we reported, since sensitivity and specificity are not impacted by disease prevalence. However, a larger sample size would have likely allowed us to narrow the confidence intervals around our ROC areas, improving the precision of the estimates.

**Table 2:** Missing data due to sample limitations

Test (n)	Sample type	Number of insufficient/indeterminate samples	Total number of samples analyzed
Cobas® DNA (77)	FNA	6	71
	OP Swab	2	75
	Saliva	4	73
Cervista® HR (37)	FNA	5	32
	OP swab	15	22
Cervista® HPV Genotype (37)	FNA	6	31
	OP Swab	14	23

## Conclusion

This study shows that it is possible and feasible to accurately diagnose oncogenic HPV infection of the oropharynx through non-invasive surrogate testing. We recommend future studies to focus on the validation of such diagnostic tests on the general population, as well as on patients with CUP for improved risk stratification in these patient groups.

## Abbreviations

AUC: Area under the curve
CUP: Cancer of unknown primary
DNA: Deoxyribonucleic acid
HPV: Human papilloma virus
FNA: Fine needle aspirate
OP: Oropharyngeal
OPSCC: Oropharyngeal squamous cell carcinoma
ROC: Receiver operating characteristics
